# Preparing for PrEP: Perceptions and Readiness of Canadian Physicians for the Implementation of HIV Pre-Exposure Prophylaxis

**DOI:** 10.1371/journal.pone.0105283

**Published:** 2014-08-18

**Authors:** Malika Sharma, James Wilton, Heather Senn, Shawn Fowler, Darrell H. S. Tan

**Affiliations:** 1 Division of Infectious Diseases, University of Toronto, Toronto, Ontario, Canada; 2 Canadian AIDS Treatment Information Exchange, Toronto, Ontario, Canada; 3 Faculty of Medicine, University of Toronto, Toronto, Ontario, Canada; 4 Hassle Free Clinic, Toronto, Ontario, Canada; 5 Division of Infectious Diseases, St Michael's Hospital, Toronto, Ontario, Canada; McGill University Health Centre, McGill University, Canada

## Abstract

Recent evidence has demonstrated the efficacy of pre-exposure prophylaxis (PrEP) for HIV prevention, but concerns persist around its use. Little is known about Canadian physicians' knowledge of and willingness to prescribe PrEP. We disseminated an online survey to Canadian family, infectious disease, internal medicine, and public health physicians between September 2012–June 2013 to determine willingness to prescribe PrEP. Criteria for analysis were met by 86 surveys. 45.9% of participants felt “very familiar” with PrEP, 49.4% felt that PrEP should be approved by Health Canada, and 45.4% of respondents were willing to prescribe PrEP. Self-identifying as an HIV expert (odds ratio, OR = 4.1, 95% confidence interval, CI = 1.6–10.2), familiarity with PrEP (OR = 5.0, 95%CI = 1.3–19.0) and having been asked by patients about PrEP (OR = 4.0, 95%CI = 1.5–10.5) were positively associated with willingness to prescribe PrEP on univariable analysis. The latter two were the strongest predictors on multivariate analysis. Participants cited cost and efficacy as major concerns. 75.3% did not feel that information had been adequately disseminated among physicians. In summary, Canadian physicians demonstrate varying levels of support for PrEP and express concerns about its implementation. Further research on real-world effectiveness, continuing medical education, and clinical support is needed to prepare physicians for this prevention strategy.

## Introduction

Curbing the incidence of new HIV infections is an important public health goal in Canada and globally. Recent studies demonstrate the efficacy of pre-exposure prophylaxis (PrEP), or the use of antiretroviral medications by HIV-negative persons to prevent HIV acquisition in high-risk settings. The iPrEx trial, a randomized placebo-controlled trial of 2499 HIV-negative men who have sex with men (MSM) and transgender women, found daily oral tenofovir and emtricitabine (TDF/FTC) 44% effective in reducing incident HIV infection, with efficacy rising to 73% among those reporting >90% adherence. [1] TDF/FTC was associated with a 75% reduction in HIV acquisition among serodiscordant heterosexual couples in a large Sub-Saharan African population (Partners PrEP), and a 62.6% reduction was seen in heterosexual transmission in Botswana (TDF2). [2], [3] Efficacy in TDF2 rose to 78% when followup data was censored 30 days after the last reported dose of PrEP to reflect adherence. Recent data suggests that daily oral TDF is also effective in preventing HIV acquisition among injection drug users. [4] In contrast, TDF/FTC was ineffective in preventing HIV acquisition among African women in both the Fem-PrEP and VOICE trials, findings likely driven by poor adherence among study participants. [5]–[7] Oral TDF/FTC was licensed for use as PrEP by the United States Food and Drug Administration on July 16, 2012.

In addition to concerns regarding adherence, anxieties have been raised regarding PrEP safety, tolerability, development of drug resistance, cost-effectiveness and risk compensation, or changes in behaviour related to a perceived decrease in risk. [8]–[13] Surveys of MSM in North America suggest that while many would consider taking PrEP to reduce transmission, overall knowledge of PrEP has been low. [10], [14]–[18] The Centers for Disease Control (CDC) in the U.S. and the Ministry of Health and Social Services in Quebec have each released interim guidelines on the use of TDF/FTC for PrEP among MSM and heterosexual adults, but clinical implementation is complex and not yet widespread. [19]–[22] Several studies have evaluated healthcare provider and physician perspectives on PrEP, but none have evaluated the Canadian perspective and sampled a broad base of generalist and specialized potential PrEP providers. [23]–[29] In Canada, physicians will likely be the sole prescribers of PrEP, as well as primary sources of PrEP information, but little is known about their knowledge of and willingness to prescribe PrEP. Understanding their perceptions, readiness, and learning needs is thus essential for safe and effective PrEP prescribing.

## Methods

We conducted a 34-item anonymous online survey of Canadian physicians using the Fluid Surveys website between September 28 2012 and June 19 2013. (Appendix S1) Canadian physicians likely to provide PrEP (i.e. those self-identifying as family practitioners, public health practitioners, infectious disease specialists and internists) were eligible for participation. A convenience sample of participants was recruited by email through the listservs of relevant physician organizations (Association of Medical Microbiology and Infectious Diseases Canada, Canadian Public Health Association, the Association of Ontario Health Centres, CIHR Canadian HIV Trials Network, and Canadian Society of Internal Medicine), primary care clinics known to serve HIV-positive and at-risk populations; personal networks, and Canadian HIV physician websites (HIV virtual medzone, viroXchange). Convenience sampling was used because the primary goal of this study was to ascertain the opinions and beliefs of Canadian physicians most likely to be involved in PrEP prescribing should it become approved for use in Canada. Physicians in training (e.g. medical students, residents, and fellows) were excluded. No prior knowledge of PrEP was required for participation. Participants were entered into a draw to win an iPad or one of 5 $20 bookstore gift certificates.

The survey instrument was designed based on a review of published literature regarding PrEP, with particular attention to key areas of uncertainty or controversy. The four domains covered in the survey included a) demographic and practice-related information, b) knowledge of and experience with PrEP, c) opinions about PrEP, and d) PrEP-related learning needs. Questions included multiple choice, Likert scale and open-ended formats.

Our primary objective was to assess physician willingness to prescribe PrEP, defined as responding ‘yes’ to the question, “Knowing what you know about PrEP now, would you prescribe PrEP for a patient at high risk of HIV infection, if they had a mechanism to cover the medication costs?” Secondary objectives were to estimate levels of physician support for regulatory approval of PrEP in Canada, physician PrEP-related knowledge, patient-initiated questioning about PrEP, and PrEP prescribing. We also sought to determine whether and how patients were obtaining off-label PrEP. Lastly, we inquired about physician beliefs around PrEP implementation issues including efficacy, target patient populations, payment mechanisms, cost-effectiveness, side effects, risks, and knowledge gaps. Relevant clinical trial data was provided within the survey with the following statement: “According to one study among men who have sex with men (MSM), oral PrEP provided 44% protection against HIV infection overall and 73% protection in participants who used PrEP consistently (i.e. took the medication on a regular schedule and did not miss doses). Other studies show that PrEP provides a similar level of protection among heterosexual men and women.” A concise description of PrEP was provided. Six pilot participants tested the survey for clarity, and feedback was incorporated into the final version.

Quantitative data was summarized using measures of central tendency, frequencies and proportions, while qualitative responses to open-ended questions were analyzed for common themes. In an exploratory analysis, a multivariable logistic regression model was built to identify respondent characteristics associated with willingness to prescribe PrEP using backwards selection. The purpose of this exploratory analysis was to help understand how opinions on this controversial emerging intervention are diffusing among physicians, rather than to precisely quantify these relationships. Variables initially considered for inclusion in the model were selected based on plausibility, and included sex, being an infectious diseases physician, working in an academic setting, proportion of professional time devoted to clinical work, self-identifying as an HIV expert, having previously been asked by a patient about PrEP, level of familiarity with PrEP, years spent in practice, and proportion of patients served who were HIV-positive. After removal of variables due to collinearity, additional variables were removed one at a time until all remaining variables made partial contributions to predicting the primary outcome using an alpha = 0.10 significance threshold. Because participants were not enrolled on the basis of specific physician characteristics, variables were only included in the model where numbers permitted. Statistical analyses were conducted using SAS version 9.3.

Our target sample size was calculated based on the minimum required number of participants to estimate the level of support for PrEP, expressed as the proportion willing to prescribe PrEP. Because there were no published data on this proportion at the time of study initiation, we conservatively estimated the true proportion at 0.5. Using the equation N = (Z_1-α/2_)^2^ * p (1-p)/l^2^, where Z_1-α/2_ is the 1-α/2 critical value of the standard normal distribution, p is the proportion of interest, and l is the length of the desired 95% confidence interval, the required sample size to allow estimation of the true prevalence, p ±0.1 was estimated at 97 participants.

The study protocol was approved by the St. Michael's Hospital Research Ethics Board. An electronic letter of information about the study preceded the survey, and participants' completion of the survey was considered to constitute implied consent. Participants could withdraw at any point during the survey.

## Results

A total of 104 responses were received, of which 86 had responses for the primary outcome and were included in the final analysis. The broad, convenience-sampling based strategy for survey dissemination did not permit estimation of response rate. Table 1 summarizes demographic characteristics, baseline knowledge, and experience with PrEP. Most participants were either infectious diseases specialists (51.1%) or general practitioners (40.5%), identified as experts in HIV care (53.5%), and practiced in an academic setting (61.2%). Though most respondents were from Ontario (61.2%), there was representation from all Canadian regions except the Territories. Respondents had been in practice for a median of 11.5 years (interquartile range, IQR 5-20), and 52.3% spent more than half their time doing clinical work. Most served substantial numbers of persons from high-risk populations, including MSM, people from HIV-endemic countries, and injection drug users.

**Table 1:** Respondent demographics.

Variable		N = 86^a^
Sex^b^		
	Male	45 (52.9)
	Female	40 (47.1)
Specialty		
	General Practice	34 (40.5)
	General Internal Medicine	4 (4.8)
	Infectious Diseases	43 (51.1)
	Medical Microbiology	1 (1.2)
	Infectious Diseases/Microbiology	1 (1.2)
	Public Health Specialist	1 (1.2)
Predominant type of practice setting		
	Private	14 (16.5)
	Community	6 (7.1)
	Academic	52 (61.2)
	Community Health Centre	6 (7.1)
	Sexual Health Clinic	4 (4.7)
	Walk-in Clinic	1 (1.2)
	Public Health	2 (2.4)
Region of Practice		
	British Columbia	7 (8.2)
	Prairies	8 (9.4)
	Ontario	52 (61.2)
	Quebec	11 (12.9)
	Atlantic	7 (8.2)
Years in Practice		11.5 (5,20)
More than 50% of time spent on clinical work		45 (52.3)
Proportion HIV-positive patients		10 (2,40)
Proportion HIV-negative patients at high risk of acquisition		10 (5,20)
Self identified expert in HIV care		46 (53.5)
Physicians serving substantial populations of following high-risk individuals		
	People from HIV-endemic countries	59 (95.2)
	MSM	55 (90.2)
	Intravenous drug users	46 (90.2)
	First Nations populations	28 (84.9)
	Commercial sex workers	27 (73.0)
	Incarcerated individuals	17 (65.4)

a Responses may not sum to 86 due to missing values.

b Values shown are number (percentage) or median (IQR).

Familiarity with PrEP was good, with 45.9% indicating they were very familiar with PrEP (“I am aware of the results of recent trials”) 37.7% somewhat familiar, and 16.5% not at all familiar (“this is my first time hearing about it”). Within the last year, 32.6% had been asked about PrEP, on a median of 2 occasions (IQR 2–10), predominantly by MSM (71%) or serodiscordant couples (60.9%). A sizeable minority (12.9%) had ever prescribed PrEP, on a median of 2 occasions in the past year (IQR 1–4), again predominantly to MSM (38.9%) or serodiscordant couples (31.3%). Eighteen physicians answered the question “If any of your patients have used PrEP off-label, do you know how they obtained it?” Responses included physician prescription (n = 4), HIV-positive partners or friends (n = 2), online purchasing (n = 1), other informal channels (n = 3), or unsure (n = 8).

**Figure 1:**
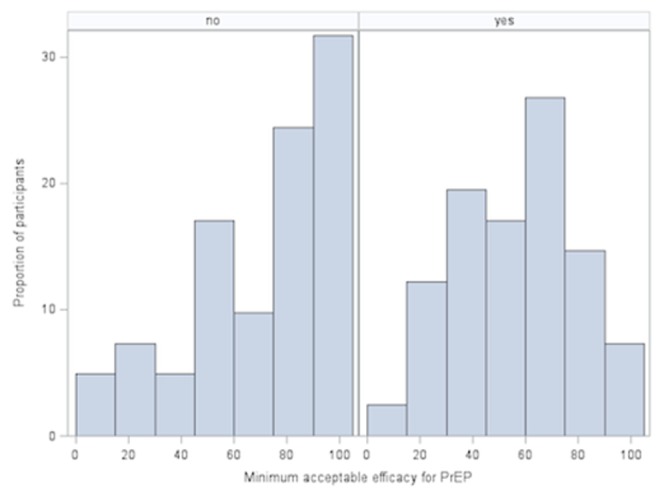
Minimum acceptable level of protection afforded by (PrEP) according to participant opinion on Health Canada Approval of PrEP in Canada. Histogram demonstrating the distribution of minimum acceptable PrEP efficacy according to whether study participants believe that PrEP should be approved for use by Health Canada. Respondents were asked “What is the MINIMUM level of protection you would consider reasonable for PrEP use to be recommended to individuals at high risk of HIV infection?” Respondents were then divided based on whether they answered yes (‘‘yes’’) or no or maybe (‘‘no’’) to the question: “According to one study among MSM, oral PrEP provided 44% protection against HIV infection overall and 73% protection in participants who used PrEP consistently (i.e. took the medication on a regular schedule and did not miss doses). Other studies show that PrEP provides a similar level of protection among heterosexual men and women. Considering this level of protection, do you believe Health Canada should approve PrEP for use in Canada?’’

The primary analysis revealed that just under half of participants (45.4%) were willing to prescribe PrEP, while 4.7% were unwilling and 50% were unsure (Table 2). Respondents' modest willingness to prescribe PrEP was mirrored by modest levels of support for regulatory approval of PrEP; 49.4% believed that PrEP should be approved by Health Canada, 37.4% responded “maybe”, while 13.3% felt it should not be approved. Overall, the median minimum efficacy considered reasonable for PrEP implementation among high-risk individuals was 66%, ranging from 10 to 100% (IQR 40–80%). As might be expected, participants who felt PrEP should be approved for use were willing to accept a lower PrEP efficacy (median 50%, IQR 40–70%) than those who were unsure or felt that it should not be approved (median acceptable efficacy 75%, IQR 50–90%) (p  =  0.004, Wilcoxon two-sample test; Figure 1).

**Table 2:** Familiarity with and attitudes regarding PrEP^a^.

Variable		N = 86^b^
Familiarity with PrEP		
	Not at all familiar	14 (16.5)
	Somewhat familiar	32 (37.7)
	Very familiar	39 (45.9)
Have been asked about PrEP in the last year		28 (32.6)
	Number of occasions	2 (2,10)
Category of patient inquiring about PrEP		
	MSM	22 (71.0)
	Serodiscordant couple	14 (60.9)
	Commercial sex worker	1 (8.3)
	Intravenous drug user	1 (8.3)
Have ever prescribed PrEP		11 (12.9)
	Number of occasions in past year	2 (1,4)
Category of patients to whom PrEP was prescribed		
	MSM	7 (38.9)
	Serodiscordant couple	5 (31.3)
	Commercial sex worker	1 (8.3)
	Other (“Pregnancy attempt”)	1 (8.3)
Enthusiasm if ever discussed PrEP with a patient		
	Unenthusiastic	3 (3.5)
	Neutral	25 (29.1)
	Enthusiastic	8 (9.3)
	Not applicable	50 (58.1)
Proportion willing to prescribe PrEP^c^		
	Willing	39 (45.5)
	Unwilling	4 (4.7)
	Unsure	43 (50)
Minimum acceptable level of protection provided by PrEP (%) for it to be recommended to high-risk individuals		66 (40,80)
Belief that Health Canada should approve PrEP for use in Canada^d^		
	Yes	41 (49.4)
	No	11 (13.3)
	Maybe	31 (37.4)

a Values shown are number (percentage) or median (IQR).

b Responses may not sum to 86 due to missing values.

c Proportion of respondents willing to prescribe PrEP based on current knowledge for high-risk patients who have a mechanism to cover medication costs.

d Response to question “According to one study among men who have sex with men (MSM), oral PrEP provided 44% protection against HIV infection overall and 73% protection in participants who used PrEP consistently (i.e. took the medication on a regular schedule and did not miss doses). Other studies show that PrEP provides a similar level of protection among heterosexual men and women. Considering this level of protection, do you believe Health Canada should approve PrEP for use in Canada?”

Factors associated with willingness to prescribe PrEP on univariable analysis are summarized in Table 3. Among the variables associated with willingness to prescribe PrEP on univariable analyses were having previously prescribed PrEP (OR = 7.0, 95%CI = 1.4–34.6) and believing that one's patients would benefit from PrEP (OR = 6.5, 95%CI = 1.2–34.0), confirming the internal consistency of the data obtained by this study. On exploratory multivariable analysis, high familiarity with PrEP (adjusted OR = 4.0, 95%CI = 1.5–10.6) and having been asked about PrEP (aOR = 2.6, 95%CI = 0.9–7.4) were the strongest predictors of willingness to prescribe, as opposed to any demographic or fixed practice-related characteristics.

**Table 3:** Factors associated with willingness to prescribe PrEP.

Variable		Univariable Analysis		Multivariable Analysis	
		OR (95% CI)	p-value	OR (95% CI)	p-value
Sex					
	Female	1.0			
	Male	2.3 (1.0–5.6)	0.06		
Specialty					
	All others	1.0			
	Infectious diseases	1.3 (0.6–3.1)	0.52		
Practice Type					
	Non-academic	1.0			
	Academic	1.1 (0.5–2.6)	0.85		
Self-identified HIV expert					
	No	1.0			
	Yes	4.1 (1.6–10.2)	0.002		
Familiarity with PrEP					
	Not familiar	1.0		1.0	
	Somewhat familiar	1.0 (0.2–3.9)			
	Very familiar	5.0 (1.3–19.0)	0.002	4.0 (1.5–10.6)	0.005
Ever been asked about PrEP					
	No	1.0		1.0	
	Yes	4.0 (1.5–10.5)	0.004	2.6 (0.9–7.4)	0.07
Ever prescribed PrEP					
	No	1.0			
	Yes	7.0 (1.4–34.6)	0.008		
Thinks his/her patients would benefit from PrEP					
	No	1.0			
	Maybe	1.9 (0.3–10.8)			
	Yes	6.5 (1.2–34.0)	0.01		
Proportion of time spent in clinical work (per 10% increase)		0.9 (0.8–1.1)	0.36		
Years in practice (per decade)		1.5 (1.0–2.2)	0.07		
Proportion of HIV-positive patients (per 10% increase)		1.1 (1.0–1.3)	0.07		
Proportion of HIV-negative patients (per 10% increase)		1.0 (0.8–1.3)	0.74		

**Figure 2:**
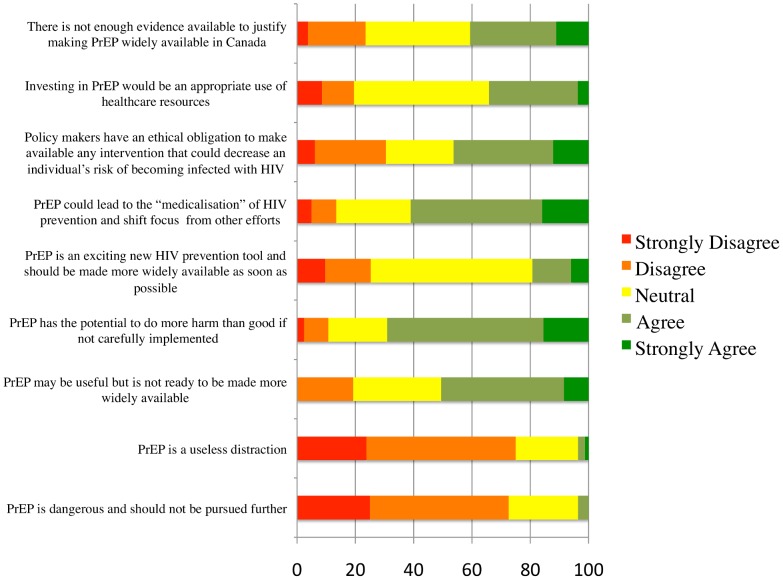
Physician perceptions of PrEP.

To assess respondents' opinions on PrEP, participants were asked to rank their level of agreement with a series of statements using a five-point Likert Scale (Figure 2). Although very few endorsed the most negative statements about PrEP (“PrEP is dangerous and should not be pursued further” and “PrEP is a useless distraction”), respondents generally voiced caution regarding PrEP rollout, with 69.1% of participants agreeing or strongly agreeing that “PrEP has the potential to do more harm than good if not carefully implemented”.

**Figure 3:**
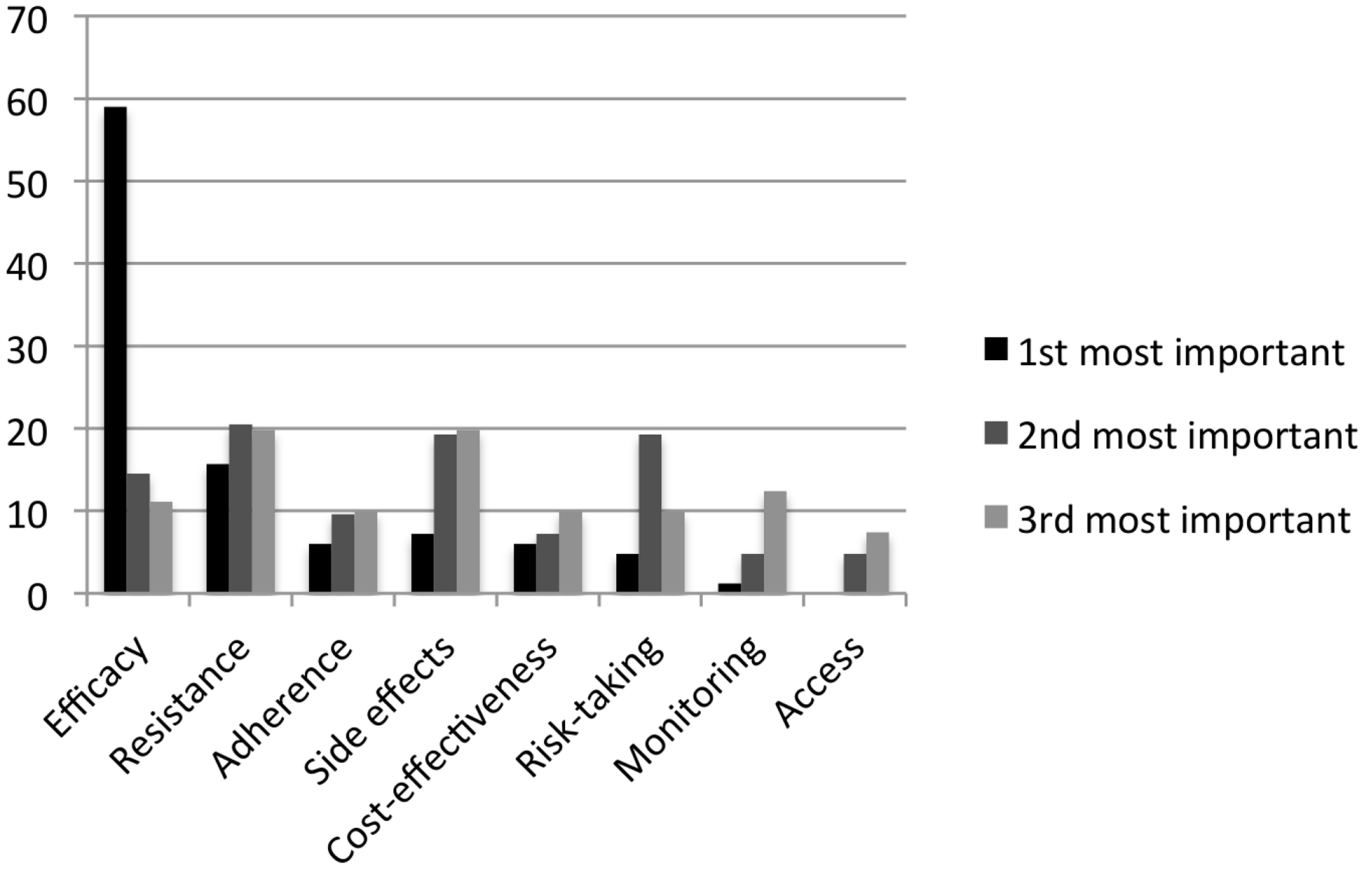
Considerations in the implementation of PrEP ranked by order of importance. Bars represent percentage of respondents.

Participants were asked to what extent their opinions and beliefs regarding whether or not PrEP should be made widely available in Canada were shaped by a series of issues drawn from existing literature. [8]–[12], [24], [25], [30], [31] The most commonly cited concern by far was PrEP's level of efficacy, followed by the potential for drug resistance and side effects (Figure 3). The risk of patients not adhering to necessary monitoring and cost-effectiveness were also frequently ranked among the top three concerns by participants.

Respondents felt that PrEP delivery could occur in a number of different settings: dedicated PrEP clinics (84.0%), sexually transmitted infection or HIV clinics (97.3%), or any physician office (81%). However, only 46.8% agreed or strongly agreed that they had enough current knowledge about PrEP to make informed prescribing decisions, while 39.0% disagreed or strongly disagreed with this statement. A variety of sources were cited as potentially useful for learning more about PrEP, most commonly including continuing medical education (CME) events (91.5%), journal articles (87.3%), online modules (84.1%), and online resources (81%). Participants' major barriers to prescribing PrEP were drug costs (91.9%), insufficient data (81.1%), unfamiliarity (77.5%), lack of clarity regarding which patient populations were appropriate for PrEP (60.7%), and a perception that patients are uninterested in PrEP (50%). When asked what supports would be needed before implementing PrEP, 92.2% cited nursing support, 89.8% cited social work support, and 89.5% cited the need for CME. Qualitative responses to the question of what is needed before PrEP is made available as an HIV prevention strategy highlighted the need for more education of recipients, providers, pharmacists, the general public, insurers, and government, with clear guidelines for prescribing and monitoring. A common demand was for more data on the effectiveness of PrEP in real-world conditions and on who is most likely to benefit from PrEP. Several participants commented on the need for improved overall HIV prevention strategy and expressed concern that funding of PrEP programs could potentially detract from other preventive strategies. The need for cost-effectiveness data was also frequently cited.

## Discussion

Although there are currently no plans to pursue regulatory approval of oral TDF/FTC as PrEP in Canada, off-label use in Canada is likely to increase in the future as community awareness increases and as real-world implementation data are reported from community-based studies and ongoing PrEP demonstration projects worldwide. [32]–[34] Indeed, in our study conducted in 2012 and early 2013, 12.9% of respondents had already prescribed PrEP in the last year, and 20.9% reported knowledge of patients using PrEP off-label. Because infectious diseases, internal medicine, public health and primary care physicians will likely be a primary source of information regarding PrEP, as well as the sole prescribers of PrEP, it is important to understand the knowledge, opinions and learning needs of these physicians to inform policy and practice decisions.

Our study found modest levels of support for PrEP among such physicians in Canada, with 45.5% willing to prescribe it and 49.4% believing that Health Canada should approve its use in Canada. Our group has also documented similar levels of cautious enthusiasm for PrEP among Canadian AIDS service organization workers and pharmacists. [35], [36] Physicians' moderate enthusiasm about PrEP was tempered by concerns described in other literature on PrEP, including imperfect efficacy, the potential for drug resistance, adverse events, cost, and the potential for risk compensation.[8]–[12], [24], [25], [27], [30], [31]

Not surprisingly, participants expressing support for Health Canada approval of PrEP were more willing to prescribe PrEP. Those expressing support for regulatory approval were also more likely to accept a lower minimum efficacy of PrEP (median 50%, IQR 40–70%) compared to those who did not think that PrEP should be approved (median 75%, IQR 50–90%). Efficacy was ranked as the most important concern about PrEP by the majority of participants (Figure 3). Of note, some clinical trial data suggests an efficacy of 73% among highly adherent MSM and 75% in serodiscordant heterosexual couples, which is comparable to the minimum acceptable efficacy even among those expressing that PrEP should not be approved. [1], [2] Taken together, these findings suggest that improving medication adherence could be pivotal to increasing the endorsement of PrEP by Canadian prescribers. As such, it is encouraging that novel strategies for supporting PrEP adherence such as text message reminders are currently being evaluated. [37] Recent pharmacologic data suggests that efficacy levels as high as 96% may be achieved when PrEP is dosed as little as four times per week are also encouraging in this regard. [38]

Exploratory logistic regression analysis demonstrated that willingness to prescribe PrEP was significantly associated with being a self-described HIV expert, having high baseline familiarity with PrEP, and having previously been asked by a patient about PrEP; the latter two were the strongest predictors in exploratory multivariable analysis. These results are consistent with data from predominantly non-physician healthcare providers in the United States showing that practitioners with higher greater knowledge of PrEP were more likely to prescribe it. [25] Interestingly, demographic and practice characteristics were not found to be important in our study. In particular, region of practice was not associated with willingness to prescribe PrEP, despite the differences in the characteristics of the HIV epidemic in different parts of Canada. These observations suggest that to implement PrEP more broadly in Canada, there is a need for increased physician education and patient dialogue with their care providers about PrEP.

A variety of physician needs for supporting PrEP rollout are also highlighted by our data. First, several respondents called for a clear set of guidelines around target populations, prescribing and monitoring of PrEP. It is noteworthy in this regard that PrEP Guidelines have since been published in Quebec; we did not assess awareness of the earlier CDC guidelines in our study. [19]–[21] Second, the majority of participants expressed interest in receiving CME through a variety of media. That only 45.9% of our sample felt very familiar with PrEP, while fully 32.6% had been asked by patients about it in the past year further supports the need for increased physician education. Third, respondents endorsed a need for more human resources such as nursing and social work supports in their clinical practices. However, we did not inquire about the specific ways in which they might be helpful.

There are several limitations to our study. First, our sample size was small and based on convenience sampling, with over-representation of academic physicians. It is difficult to determine the impact of having a largely academic sample on our findings. We were unable to calculate a response rate due to the multi-modal nature of participant recruitment through listservs, websites, and professional organizations with overlapping membership. To our knowledge, however, this is the first study to provide data on a sample of general healthcare providers in Canada who are likely to prescribe or counsel about PrEP. Other Canadian data has focused solely on infectious diseases practitioners. [27] In addition, given our study objectives, we sought to reach a sample of Canadian physicians that was likely to be engaged in PrEP delivery and the debate over its delivery in the future, based on existing familiarity with HIV medicine. Second, we conducted our survey at a time when new data on PrEP were continually emerging, and a single survey can only provide a snapshot of physician opinions at any given point in time. For instance, the negative results of the VOICE trial were reported during the study period, and it was not possible to assess whether this information affected participants' views. Similarly, a large study reporting positive results among intravenous drug users in Thailand was published after our study period, and the lack of data on this population during the study period may have contributed to our participants' expressed concerns that it was unclear which populations would benefit most from PrEP. Finally, the use of an electronic survey instrument may have selected for younger physicians or those with increased familiarity with electronic media. As with any survey study, there is also the potential for differential responses to questions as a result of social desirability bias.

Should PrEP be widely implemented in Canada, physicians will be at the front-lines of patient education, prescription, and monitoring. As such, assessing their perceptions and learning needs around PrEP is an essential part of the stakeholder evaluation before widespread roll-out of this prevention tool. As further research emerges on real-world effectiveness, novel formulations of PrEP, and outcomes in different populations, ongoing support for both patients and physicians will be needed to optimize the clinical and public health impact of this important HIV prevention strategy.

## Supporting Information

Appendix S1 **Health Care Providers Survey (English).**
(PDF)
