# Supplementary material for: Preparing for PrEP: Perceptions and Readiness of Canadian Physicians for the Implementation of HIV Pre-Exposure Prophylaxis
Source: PLoS One. 2014 Aug 18;9(8):e105283. doi: 10.1371/journal.pone.0105283 (PMC4136811; doi:10.1371/journal.pone.0105283)
Supplement: Appendix S1 — Health Care Providers Survey (English). (PDF) [file pone.0105283.s001.pdf]

Physician PrEP Survey

Preparing for PrEP: Study details

LETTER OF INFORMATION REGARDING STUDY

STUDY TITLE
Assessing perceptions, beliefs, and readiness of Canadian physicians for the implementation of HIV pre-exposure prophylaxis.

INVESTIGATORS
Darrell Tan, MD FRCPC
Malika Sharma, MD FRCPC
Heather Senn, MSc
James Wilton, CATIE
Contact: (416) 864-5568, prepsurvey@gmail.com

INTRODUCTION
You are being asked to participate in a nation-wide research study being conducted by a research team from St. Michael's Hospital in Toronto. Before agreeing to participate in this study, it is important that you read and understand the following explanation of the study. You are free to accept or decline participation in the study without consequence.

PURPOSE OF THE STUDY
This questionnaire has been designed to find out about physician knowledge, opinions and learning needs regarding pre-exposure prophylaxis (PrEP) for prevention of HIV infection. This project will have important implications for public health and the availability of services to people at risk of getting HIV infection. You do not need to have any prior knowledge of PrEP to participate in the survey.

STUDY PROCEDURES
• Participation in this study is voluntary. If you agree to participate, you will be asked to fill out this six page online questionnaire.
• This survey is confidential. Please do not include your name or any other identifying information other than what is asked in the survey.
• This survey takes about 15-20 minutes to complete and asks you questions about your knowledge and experience with PrEP, your opinions on key issues and your learning needs.
• You have the option to not answer any questions which you find confusing, uncomfortable or choose to omit for any reason.
• You also have the option to withdraw your consent by not completing the questionnaire if you so wish for any reason. If you choose to withdraw, clicking on the "discard responses and exit" button will delete all your responses and close the survey.
• Once your responses have been submitted you will not be able to withdraw from the study. Since all responses are anonymous and confidential it will be impossible to identify your questionnaire responses.
• At the end of the survey you will be given the opportunity to provide your email address (via a separate link) for a chance to win an iPad or one of five $20 gift certificates to Chapters/Indigo. Your email address will not be linked in any way to your survey responses.

CONFIDENTIALITY
All information obtained during the study will be held in strict confidence. Completed surveys will be securely stored until study completion, after which the surveys will be securely destroyed. Unless otherwise required by law, access to study surveys will be limited to the study investigators and their delegates, the St. Michael's Hospital Research Ethics Board for the purpose of monitoring the study, and the survey provider (FluidSurvey). The results of this study may be presented by the study team at conferences, seminars or other public forums, and published in journals.

POTENTIAL RISKS
There are no known risks associated with participation in this study.

POTENTIAL BENEFITS
Benefits to you from participating in this study may include gaining some knowledge of PrEP and issues surrounding its use. Other health care providers who serve populations at risk for HIV may also benefit from your participation in this study in the future. Upon completion of the survey, you may choose to have your name entered into a draw to win an iPAD or Chapters Indigo gift certificate.

INFORMED CONSENT

By clicking the box below, you are agreeing that you have read and understood the informed consent form and agree to participate in the study. If you require further information about this study, you may contact Dr. Darrell Tan, Principal Investigator, at 416-864-5568 during business hours. This study has been approved by the Research Ethics Board at St. Michael's Hospital. If you have any questions regarding your rights as a research participant, you may contact the Office of Research Ethics at 416-864-6060 Ext. 2557.

☐ I have read and understood the detailed consent form and agree to participate in the study

1. What are the first three characters of your practice's postal code?

First character ____________________

Second Character ____________________

Third Character ____________________

2. Do you identify as:

- Male
- Female
- Trans Man (F to M)
- Trans Woman (M to F)
- None of these describe me (specify) ________________________

3. What type of physician are you?

- General practitioner/family physician
- Infectious diseases specialist
- General internist
- Other, please specify: ________________________

4. In what type of setting do you practice predominantly?

- Private practice
- Community hospital
- Academic hospital
- Community health centre
- Walk-in clinic
- Sexual health clinic
- Other, please specify: ________________________

5. What percent of your work time do you spend on the following activities?

Clinical ____________________

Administrative ____________________

Research ____________________

Public Health ____________________

Teaching ____________________

6. I identify as a specialist in HIV care:

- Strongly Disagree
- Disagree
- Neutral
- Agree
- Strongly Agree

7. How many years have you been working in independent clinical practice (i.e. after completing residency)?:

____

8. What proportion of your patient population is HIV-positive (please enter an estimated percentage)?

____

9. What proportion of your patient population is HIV-negative but at high risk for HIV-acquisition (please enter an estimated percentage)?

10. Does your practice serve a substantial population of any of the following groups? (check all that apply)

- [ ] Aboriginal peoples
- [ ] Gay, bisexual and other men who have sex with men, and trans communities
- [ ] People from countries where HIV is endemic and people from ethnoracial/racialized communities
- [ ] People who use injection drugs
- [ ] Prison inmates
- [ ] Sex workers and their clients
- [ ] Women

Branching Information
• If (10 = Not familiar at all (eg. this is my first time hearing about it)) or (10 has no response) then Hide 11

Pre-exposure prophylaxis for HIV prevention

PrEP is a new HIV prevention method that has recently been found effective in clinical trials. It involves an HIV-negative individual taking anti-retroviral drugs in an effort to reduce their risk of becoming infected with HIV. Using the current strategy, PrEP drugs (typically tenofovir and/or emtricitabine) need to be taken daily on a regular basis —starting before and continuing after exposure to HIV.

Clinical trials show that PrEP is only partially protective against HIV infection and much less so if not taken consistently. People using PrEP would need to commit to regular doctor's appointments to monitor adverse effects and adherence. Patients would also require regular testing for HIV and other sexually transmitted infections. PrEP has not been approved by Health Canada and is not yet widely available.

In this survey, PrEP specifically refers to the daily use of anti-retroviral pills by people who are HIV-negative, also known as oral PrEP. PrEP is not the same as post-exposure prophylaxis or PEP, which is the daily use of antiretroviral pills for 28 days AFTER a potential exposure.

11. How would you describe your current knowledge about PrEP?(10)

- ◯ Not familiar at all (eg. this is my first time hearing about it)
- ◯ Somewhat familiar (eg. I am aware of PrEP and the existence of clinical trials but not of their details)
- ◯ Very familiar (eg. I am aware of the details of recent clinical trials)

12. Where did you hear about PrEP? (Check all that apply)(11)

- ☐ Peer-reviewed medical journal
- ☐ HIV/AIDS-related or other medical conference
- ☐ Workshop, lecture or seminar
- ☐ Colleague
- ☐ Client/Patient
- ☐ Website or blog
- ☐ Newspaper or magazine
- ☐ Other, please specify: ________________________

13. Do you have any thoughts/opinions/concerns on the use of oral PrEP for the prevention of HIV infection?

____
____
____

Branching Information
- If not 14 = Yes then Hide 14a
- If not 14 = Yes then Hide 14b
- If not 15 = Yes then Hide 15a
- If not 15 = Yes then Hide 15b

14. Have you ever been questioned by a patient about pre-exposure prophylaxis (PrEP)? (14)

- ◯ Yes
- ◯ No

If you answered yes to the previous question, how many times have you been asked in the past year?(14a)

____

Who has asked you about PrEP? (check all that apply)(14b)

- ☐ Men who have sex with men
- ☐ People in serodiscordant relationships
- ☐ Person working as a commercial sex worker
- ☐ Person using intravenous drugs
- ☐ Other, please specify: __________________________

15. Have you ever prescribed PrEP as an HIV prevention option to a patient? (15)

- ◯ Yes
- ◯ No

If you answered yes to the previous question, how many times have you prescribed PrEP in the past year? :(15a)

____

To whom have you prescribed PrEP? (check all that apply):(15b)

- ☐ Men who have sex with men
- ☐ People in serodiscordant relationships
- ☐ Person working as a commercial sex worker
- ☐ Person using intravenous drugs
- ☐ Other, please specify: __________________________

16. If you have spoken to a patient about PrEP, were you generally:

- ◯ Unenthusiastic
- ◯ Neutral
- ◯ Enthusiastic
- ◯ N/A

17. If any of your patients have used PrEP off-label, do you know how they obtained it? (Check all that apply)(17)

- ☐ Off-label prescription by a physician
- ☐ Internet purchase
- ☐ From an HIV-positive partner/friend
- ☐ Other informal channels (eg. obtained on the street)
- ☐ Unsure
- ☐ N/A

18. Knowing what you know about PrEP now, would you prescribe PrEP for a patient at high risk of HIV infection, if they had a mechanism to cover the medication costs?

- ◯ Yes
- ◯ No
- ◯ Unsure

Branching Information
- If not 22 contains Individuals with a large number of sexual partners then Hide 22a
- If not 22 contains Individuals who regularly engage in unprotected sex then Hide 22b

Opinions on PrEP implementation

19. To what extent do you agree with the following statements:

	strongly disagree	disagree	neutral	agree	strongly agree
PrEP is dangerous and should not be pursued further	◯	◯	◯	◯	◯
PrEP is a useless distraction	◯	◯	◯	◯	◯
PrEP may be useful but is not ready to be made more widely available	◯	◯	◯	◯	◯
PrEP has the potential to do more harm than good if not carefully implemented	◯	◯	◯	◯	◯
PrEP is an exciting new HIV prevention tool and should be made more widely available as soon as possible	◯	◯	◯	◯	◯

20. What is the MINIMUM level of protection you would consider reasonable for PrEP use to be recommended to individuals at high risk of HIV infection?

Please report a percentage value. For example, "x" = x % reduction in incidence of HIV infection

____

21. PrEP will likely be recommended to Individuals at high risk for HIV infection. How would you define high risk in this context? (check all that apply)(22)

- ☐ Gay, bisexual or other men who have sex with men
- ☐ Individuals with a large number of sexual partners
- ☐ Individuals who regularly engage in unprotected sex
- ☐ Individuals with a history of one or more STIs
- ☐ Individuals who have sex under the influence of drugs
- ☐ Individuals who have used post-exposure prophylaxis (PEP) before (i.e. have taken antiretroviral drugs AFTER exposure to HIV to prevent becoming infected)
- ☐ People who use injection drugs and other people who may share needles
- ☐ HIV negative individuals who are sexually active with HIV positive partners on an ongoing basis
- ☐ Individuals who exchange sex for money, goods, or housing
- ☐ Individuals belonging to any population with a high incidence of HIV
- ☐ Other, please specify: __________________________

In the context of the previous question, how would you define a large number of sexual partners? (please enter minimum number of partners per month)(22a)

____

In the context of the previous question, how would you define a regularly engaging in unprotected sex? (please enter minimum number of unprotected sexual encounters per month)(22b)

____

22. According to one study among men who have sex with men (MSM), oral PrEP provided 44% protection against HIV infection overall and 73% protection in participants who used PrEP consistently (i.e. took the medication on a regular schedule and did not

miss doses). Other studies show that PrEP provides a similar level of protection among heterosexual men and women. Considering this level of protection, do you believe Health Canada should approve PrEP for use in Canada?

- Yes
- No
- Maybe

23. PrEP medication is estimated to cost $830/month. Who do you think should pay for PrEP in Canada? (check all that apply)

- Public drug plans (the government)
- Private drug plans
- The person using PrEP (out of pocket)
- Other, please specify: ________________________

24. In what kinds of settings do you think PrEP should be prescribed, if approved by Health Canada? (check all that apply)

- Dedicated PrEP clinics
- Incorporated into STI or HIV clinics
- Individual physicians offices (like any other health issue)
- Other, please specify: ________________________

25. The following issues have been raised as important considerations for the implementation of oral PrEP. To what extent do each of these issues shape your opinions and beliefs regarding whether or not PrEP should be made widely available in Canada?

	not at all important	of little importance	neutral	somewhat important	very important
Efficacy (i.e. the % decrease in risk of HIV infection)	◯	◯	◯	◯	◯
Risk for development of antiviral drug resistance if a person using PrEP becomes infected	◯	◯	◯	◯	◯
Potential side effects and their severity	◯	◯	◯	◯	◯
Risk that PrEP use may increase risk taking (behavioural disinhibition / risk compensation)	◯	◯	◯	◯	◯
Risk that patients may not adhere to necessary monitoring and testing while taking PrEP	◯	◯	◯	◯	◯
Risk that patients may not take PrEP medications as directed, thus reducing its efficacy	◯	◯	◯	◯	◯
Cost-effectiveness of PrEP	◯	◯	◯	◯	◯
Unequal access for certain groups if funding for PrEP medications is out-of-pocket or through private insurers	◯	◯	◯	◯	◯

26. Please rank the THREE considerations you believe are the MOST IMPORTANT in order from LEAST to MOST important:

	Most important	Second most important	Third most important
The level of protection provided (i.e. the % decrease in risk of HIV infection)	☐	☐	☐
Risk for development of antiviral drug resistance if a person using PrEP becomes infected	☐	☐	☐
Potential side effects and their severity	☐	☐	☐
Risk that PrEP use may increase risk taking (behavioural disinhibition / risk compensation)	☐	☐	☐
Risk that patients may not adhere to necessary monitoring and testing while taking PrEP	☐	☐	☐
Risk that patients may not take PrEP medications as directed	☐	☐	☐
Cost-effectiveness of PrEP	☐	☐	☐

Unequal access for certain groups if funding for PrEP medications is out-of-pocket or through private insurers	☐	☐	☐

27. Do you have any other concerns that were not mentioned above but that you feel are important?

____

____

____

28. After considering the issues above, to what extent do you agree with each of the following statements?

	strongly disagree	disagree	neutral	agree	strongly agree
Policy makers have an ethical obligation to make available any intervention that could decrease an individual's risk of becoming infected with HIV	◯	◯	◯	◯	◯
PrEP could lead to the "medicalisation" of HIV prevention and take focus away from other, more important prevention efforts	◯	◯	◯	◯	◯
Investing in PrEP would be an appropriate use of healthcare resources	◯	◯	◯	◯	◯
There is not enough evidence available to justify making PrEP widely available in Canada	◯	◯	◯	◯	◯

29. Do you think some of the patients you serve would benefit from PrEP?

- Yes
- No
- Maybe

30. To what extent do you agree with each of the following statements?

	strongly disagree	disagree	neutral	agree	strongly agree
I have enough current knowledge about PrEP to make informed prescribing decisions					
I think that information about PrEP has been adequately disseminated among physicians					

31. The following media would be useful to me in learning more about PrEP: (select all that apply)

- Access to papers from peer-reviewed medical journals
- Website
- CME event or workshop
- Online learning modules
- Brochure
- N/A (I don't need more info about PrEP)
- Other, please specify: _________________________

32. What are current barriers against prescribing PrEP? (check all that apply)

- I am not familiar enough with PrEP to prescribe it
- I am unsure of which patients to prescribe it to
- I do not think my patients would be interested in PrEP as an option
- My patients are unable to get the drug costs covered
- I do not feel there are sufficient data to support its use
- Other, please specify: _________________________

33. What supports would your practice need before implementing PrEP? (check all that apply)

- Social work support to obtain drug coverage for my patients
- Continuing medical education on new evidence for PrEP
- Nursing support for ongoing patient counselling, routine STI and HIV testing and monitoring for adverse effects
- Other (please be as specific as possible) _________________________

34. What do you think is needed, in general, before PrEP is made more widely available as an HIV prevention strategy?

____
____
____

35. Any further thoughts?

____
____
____

Thank you very much for your time!

Thank you for taking the time to fill out this survey. After pressing the submit button below responses will be saved and you will no longer have the option to withdraw from the study.

If you would like to be entered in a draw to win an iPad or a gift certificate to Chapters/Indigo, copy and paste the link below into a new browser. Your email address will not be linked to your survey responses. Please also ensure that you press submit below to enter your survey responses.

http://fluidsurveys.com/s/physician-ipad-draw/
